# Blocks World of Touch: Exploiting the Advantages of All-Around Finger Sensing in Robot Grasping

**DOI:** 10.3389/frobt.2020.541661

**Published:** 2020-11-19

**Authors:** Daniel Fernandes Gomes, Zhonglin Lin, Shan Luo

**Affiliations:** ^1^smARTLab, Department of Computer Science, niversity of Liverpool, Liverpool, United Kingdom; ^2^School of Mechanical Engineering and Automation, Fuzhou University, Fuzhou, China

**Keywords:** sensor, robotics, robotic manipulation, optical tactile sensors, tactile sensing

## Abstract

Tactile sensing is an essential capability for a robot to perform manipulation tasks in cluttered environments. While larger areas can be assessed instantly with cameras, Lidars, and other remote sensors, tactile sensors can reduce their measurement uncertainties and gain information of the physical interactions between the objects and the robot end-effector that is not accessible via remote sensors. In this paper, we introduce the novel tactile sensor *GelTip* that has the shape of a finger and can sense contacts on any location of its surface. This contrasts to other camera-based tactile sensors that either only have a flat sensing surface, or a compliant tip of a limited sensing area, and our proposed GelTip sensor is able to detect contacts from all the directions, like a human finger. The sensor uses a camera located at its base to track the deformations of the opaque elastomer that covers its hollow, rigid, and transparent body. Because of this design, a gripper equipped with GelTip sensors is capable of simultaneously monitoring contacts happening inside and outside its grasp closure. Our extensive experiments show that the GelTip sensor can effectively localize these contacts at different locations of the finger body, with a small localization error of approximately 5 mm on average, and under 1 mm in the best cases. Furthermore, our experiments in a Blocks World environment demonstrate the advantages, and possibly a necessity, of leveraging all-around touch sensing in manipulation tasks. In particular, the experiments show that the contacts at different moments of the reach-to-grasp movements can be sensed using our novel GelTip sensor.

## 1. Introduction

For both humans and robots, tactile sensing is an essential capability to be exploited when performing manipulation tasks in cluttered environments. In such environments, the positions and shapes of objects are uncertain, and it is therefore of critical importance to sense and adapt to the scene. With cameras, Lidars, and other remote sensors, large areas can be assessed instantly (Peel et al., 2018). However, measurements obtained using such sensors often suffer from large inaccuracies, occlusions, and a variance of factors like light conditions. In contrast, because of the direct contact with the object, tactile sensing can reduce the measurement uncertainties of remote sensors, as it does not suffer from the aforementioned surrounding conditions. Furthermore, tactile sensing gains information of the physical interactions between the objects and the robot end-effector, for example, incipient slip, collisions, geometry, and so on—characteristics that are often not accessible via remote sensors. It is crucial to attain these accurate measurements provided by tactile sensing, as errors are less tolerable in moments of contact or near-contact. For instance, failing to grasp an object by 1 mm is more critical than failing to estimate the size of an object existing at a distance of 1 cm. To this end, camera vision and other remote sensors can be used to produce initial measurements and plan the grasp, whereas tactile sensing refines the measurement and facilitates in-hand manipulation (Luo et al., 2017).

**Figure 1:**
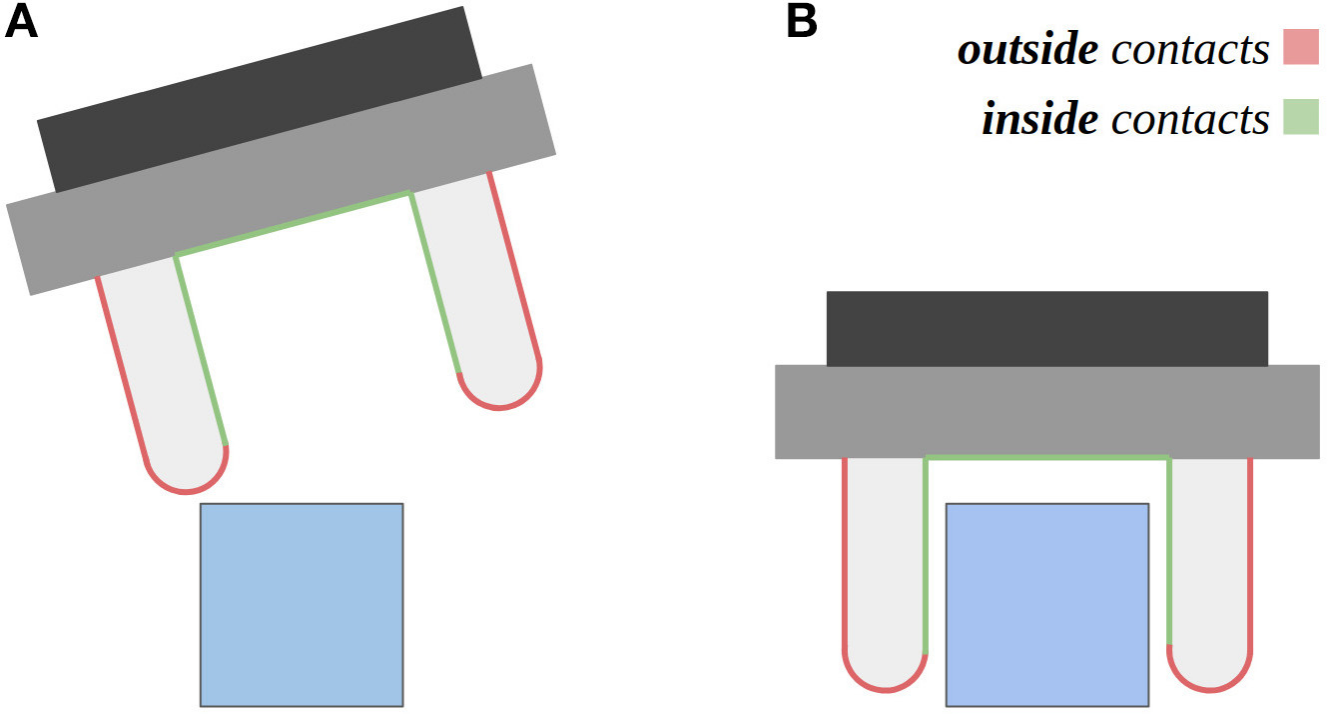
The figure depicts two distinct moments, during the execution of a manipulation task. Two distinct areas of contact are highlighted in the robot gripper: outside contacts and inside contacts in the grasp closure. **(A)** Outside contacts can be used to probe the object to be grasped, or to steer the gripper pose. **(B)** Inside contacts can be used to know when the object is within the grasp closure, and when the gripper should stop being closed.

The information provided by touch about the surfaces under contact can be leveraged for two main purposes: to perceive the properties of the contacted objects such as texture and softness, and to guide the control of motions for manipulation tasks, like detecting incipient slip while an object being grasped. While these contacts can happen over the entire robot body, in this work we focus on contacts that happen around the fingers of the gripper, as they are more actively exposed to contacts during manipulation. We can group these contacts into ones happening outside or inside of the grasp closure. As shown in Figure 1, it can be noticed that the former set of contacts is essential to detect collisions of the gripper and to probe the object to be grasped, while the latter is essential to assess the properties of the object already being, or close to be, grasped. For these reasons, the development of a tactile sensor (or a sensor suite) that is capable of covering the entire finger surface is of high importance to address robotic manipulation.

A wide range of tactile sensors have been developed in the literature (Dahiya et al., 2013; Luo et al., 2017), ranging from flexible electronic skins (Kaltenbrunner et al., 2013), fiber optic–based sensors (Xie et al., 2013), capacitive tactile sensors (Maiolino et al., 2013), to camera-based optical tactile sensors (Yuan et al., 2017; Ward-Cherrier et al., 2018), many of which have been employed to aid robotic grasping (Kappassov et al., 2015). Electronic tactile skins and flexible capacitive tactile sensors can adapt to different body parts of the robot that have various curvatures and geometry shapes. However, due to the use of dielectrics for each sensing element, they suffer from complicated electronics, cross-talk problems and low resolution of tactile signals, for example, a commercial Weiss WTS tactile sensor of 14 × 6 taxels (tactile sensing elements) used in Luo et al. (2015, 2016, 2019). Thanks to the use of cameras in the sensor, the optical tactile sensors provide high-resolution images of the deformation caused by contacts with objects in hand. The optical tactile sensors usually consist of three main parts: a soft elastomer that deforms to the shape of the object upon contact; a webcam underneath this elastomer that views the deformed elastomer; LEDs that illuminate the space between the elastomer and the webcam. There are two main families of optical tactile sensors, TacTip sensors (Ward-Cherrier et al., 2018) and GelSight sensors (Yuan et al., 2017). TacTip exploits the tracking of markers printed on a soft domed membrane, whereas GelSight exploits colored illumination and photometric stereo analysis to reconstruct the membrane deformations. Because of the different working mechanisms, TacTip only measures the surface on a few points, whereas GelSight sensors make use of the full resolution provided by the camera. However, to the authors' best knowledge, only flat surfaced GelSight sensors have been proposed so far that only have limited contact measurement areas on one side of the sensor.

To leverage the full resolution of the camera as the Gelsight sensor but also enable to detect contacts from all the directions, we propose a novel optical tactile sensor *GelTip* that has a fingertip shape and can measure contacts on any location of the fingertip surface using a camera installed in its core. The deformations of the elastomer that covers the hollow, rigid and transparent body can be captured by tracking the changes in the high-resolution outputs of the camera. In contrast with other camera-based tactile sensors, our proposed GelTip sensor is able to detect contacts from a variety of directions, including the front and side surfaces, like our human fingertip. Our extensive experiments show that our proposed GelTip sensor can effectively localize the contacts at different locations of the fingertip body, with a small localization error of 5 mm, on average, and under 1 mm in the best cases. More importantly, the results of the grasping experiments in a Blocks World environment demonstrate that the GelTip can help to gain additional information from collisions caused by inaccurate assessments from remote sensing, resulting in improved grasping policies. Such capability is only possible thanks to the all-around sensing provided by the GelTip.

The remainder of this paper is organized as follows: related works on the development of tactile sensors for robotic manipulation are reviewed and compared in section 2. A detailed introduction of our proposed GelTip sensor is presented in section 3. The results of the carried experiments are reported in section 4 and discussed in section 5. Conclusion and future directions are given in section 6.

## 2. Related Works

In contrast with remote sensors like cameras, tactile sensors are designed to assess properties of objects, for example, geometry, texture, humidity, and temperature, via physical interactions. A large range of working principles have been actively proposed in the literature (Dahiya et al., 2013; Luo et al., 2017). In this section, we focus on the set of sensors that have been used to measure the pressure/force distributions of the contact with objects in robotic manipulation, from which geometry and texture of objects can be predicted. More specifically, we compare the related works in electronic tactile skins and camera-based optical tactile sensors that have been widely used for robotic manipulation.

### 2.1. Electronic Tactile Skins

The electronic tactile skins can be grouped into five categories based on their sensing principles (Yousef et al., 2011): resistive, capacitive, piezoelectric, optical, and organic field-effect transistors (OFETs). These families of tactile sensors measure the pressure distribution of contact by the transduction of a specific electrical characteristic in response to the applied pressure on the surface of the tactile sensor. Apart from sensing and transduction principles, such tactile sensors can also be categorized by either their sensor structures (e.g., flexible printed circuit boards, extended gate transistors, and silicon transistors) (Dahiya et al., 2013) or spatial resolution and the body parts they are designed for (single-point contact sensors, high spatial resolution tactile arrays, and large-area tactile sensors) (Luo et al., 2017). Compared to camera-based optical tactile sensors, electronic tactile skins have lower thickness and are less bulkier; they can adapt to different body parts of the robot that have various curvatures and geometry shapes. However, each sensing element of most of the tactile skins (e.g., a capacitive transducer) has the size of a few square millimeters or even centimeters, which results in a limited spatial resolution of the tactile skins. For instance, a commercial Weiss WTS tactile sensor of a similar size to one adult human fingertip has only 14 × 6 taxels (tactile sensing elements) (Luo et al., 2014, 2015). In addition, they suffer from complicated electronics and cross-talk problems between neighbor sensing elements.

### 2.2. Camera-Based Optical Tactile Sensors

Camera-based optical tactile sensors make use of cameras to capture touch information. These cameras are placed at the core of an enclosed shell, pointing to an opaque window made of a soft material. Such characteristics ensure that the captured image is not affected by the external illumination variances. To extract the elastomer deformations from the captured tactile image, multiple working principles have been proposed. We group such approaches into two categories: marker tracking and raw image analysis.

One example of marker tracking-based tactile sensors is the TacTip Family of sensors described in Chorley et al. (2009) and Ward-Cherrier et al. (2018) including the TacTip, TacTip-GR2, TacTip-M2, and TacCylinder. Each TacTip sensor introduces novel manufacturing advancements or surface geometries; however, the same working principle is shared: white pins are imprinted onto a black membrane that can then be tracked using computer vision methods. In Yamaguchi and Atkeson (2016), an optical tactile sensor FingerVision is proposed to make use of a transparent membrane, with the advantage of gaining proximity sensing. However, the use of the transparent membrane makes the sensor lack the robustness to external illumination variance associated with touch sensing. Semi-opaque grids of magenta and yellow makers, painted on the top and bottom surfaces of a transparent membrane, are proposed in Lin and Wiertlewski (2019), in which the mixture of the two colors is used to detect horizontal displacements of the elastomer.

On the other side of the spectrum, the GelSight sensors, initially proposed in Johnson and Adelson (2009), exploit the entire resolution of the tactile images captured by the sensor camera, instead of just tracking makers. Due to the soft opaque tactile membrane, the captured images are robust to external light variations, and capture information of the touched surface's geometry structure, unlike most conventional tactile sensors that measure the touching force. Leveraging the high resolution of the captured tactile images, high accuracy geometry reconstructions are produced in Li et al. (2014), Luo et al. (2018), and Lee et al. (2019). In Li et al. (2014), this sensor is set as the fingers of a robotic gripper to insert a USB cable into the correspondent port effectively; however, the sensor only measures a small flat area oriented toward the grasp closure. Markers were also added to the membrane of the GelSight sensor, enabling applying the same set of methods that were explored in the TacTip sensors. There are some other sensor designs and adaptations for robotic fingers in Yuan et al. (2017), Donlon et al. (2018), and Lambeta et al. (2020). In Yuan et al. (2017), matte aluminum powder is used for improved surface reconstruction, together with the LEDs being placed next to the elastomer, and the elastomer being slightly curved on the top/external side. In Donlon et al. (2018), a mirror placed at a shallow and oblique angle is proposed for a slimmer design. The camera is placed on the side of the tactile membrane, such that it captures the tactile image reflected onto the mirror. A stretchy textured fabric is also placed on top of the tactile membrane to prevent damages to the elastomer and to improve tactile signal strength. In Lambeta et al. (2020), the authors propose a compact design, with a USB “plug-and-play” port and an easily replaceable elastomer, secured with a single screw mount.

In these previous works on camera-based optical tactile sensors, multiple designs and two distinct working principles have been exploited; however, none of the introduced sensors has the capability of assessing the entire surface of a robotic finger, that is, both the sides and the tip of the finger. As a result, these sensors are highly constrained in object manipulation tasks. Contacts are only assessed when the manipulated object is within the grasp closure (Li et al., 2014; Calandra et al., 2018; Dong et al., 2019). To address this gap, we propose the GelTip fingertip-shaped sensor. This sensor outputs tactile images captured by a camera placed in the center of a finger-shaped tactile membrane. It has a large assessed area of approximately 75 cm2 (vs. 4 cm2 of the GelSight sensor) and a high resolution of 2.1 megapixels over both the sides and the tip of the finger, with a small diameter of 3 cm (vs. 4 cm of the TacTip sensor). More details on the main differences between the GelSight sensors, TacTip sensors, and our GelTip sensor are given in Table 1.

**Table 1:** A summary of influential GelSight sensors, the TacTip family, and our GelTip sensor.

	**Sensor structure**	**Illumination**	**Tactile membrane**
**GelSight**, Li et al. (2014)	It has a cubic design with a flat square surface. A Logitech C310 (1,280 × 720) camera is placed at its base pointing at the top membrane.	Four LEDs (RGB and white) are placed at the base. The emitted light is guided by the transparent hard surfaces on the sides, so that it enters the membrane tangentially.	A soft elastomer layer is placed on top of a rigid, flat, and transparent acrylic sheet. It is painted using semi-specular aluminum flake powder.
**GelSight**, Yuan et al. (2017)	It has a close-to hexagonal prism shape. The used webcam is also the Logitech C310.	Three sets of RGB LEDs are positioned (close to) tangent to the elastomer, with a 120° angle from each other.	A matte aluminum powder is proposed for improved surface reconstruction. Its elastomer has a flat bottom and a curved top.
**GelSlim**, Donlon et al. (2018)	A mirror placed at a shallow oblique angle and a Raspberry Pi Spy (640 × 480) camera is used to capture the tactile image reflected by the mirror.	A single set of white LEDs is used. These are pointed at the mirror, so that the light is reflected directly onto the tactile membrane.	A stretchy and textured fabric on the tactile membrane prevents damages to the elastomer and results in improved tactile signal strength.
**DIGIT**, Lambeta et al. (2020)	A prismatic design, with curved sides. An OmniVision OVM7692 (640 × 480) camera is embedded in the custom circuit board.	Three RGB LEDs are soldered directly into the circuit board, illuminating directly the tactile membrane.	The elastomer can be quickly replaced using a single screw mount.
**Round Fingertip**, Romero et al. (2020)	It has a round membrane, close to a quarter of sphere. A single 160° FoV Raspberry Pi (640 × 480) is installed on its base.	Two rings of LEDs are placed on the base of the sensor, with the light being guided through the elastomer.	Both rigid and soft parts of the membrane are cast, using SLA 3D-printed molds.
**OmniTact**, Padmanabha et al. (2020)	It has a domed shape. Five endoscope cameras (400 × 400) are installed on a core mount, and placed orthogonally to each other: pointing at the tip and sides.	RGB LEDs are soldered both onto the top and sides of the sensor.	The elastomer gel is directly poured onto the core mount (and cameras) without any rigid surface or empty space in between.
**TacTip Family**,	The Tactip has a domed (finger) shape. It tracks 127 pins. More compact (TacTip-GR2) and elongated (TacTip-M2) designs have also been proposed. The TacCylinder uses catadioptric mirror to track the 180 markers around its cylindrical body.	In the TacTip sensors, a ring of white LEDs is placed at the base, around the camera sensor.	In the TacTip Family, the membrane is dark with white pins. Perception is achieved by tracking such pins. TacTip has a rounded soft membrane with a flat bottom, placed on top of a flat and rigid acrylic window.
**GelTip**, (Ours)	It has a domed (finger) shape, similar to a human finger. A Microsoft Lifecam Studio webcam (1,920 × 1,080) is used.	Three sets of LEDs, with a 120° angle from each other, are placed at the sensor base, and the light is guided through the elastomer.	An acrylic test tube is used as the rigid part of the membrane. The deformable elastomer is cast using a three-part SLA/FFF 3D-printed mold.

Two recent works Romero et al. (2020) and Padmanabha et al. (2020) also address the issue of the flat surface of previous GelSight sensors. However, their designs have large differences to ours. In Romero et al. (2020), the proposed design has a tactile membrane with a surface geometry close to a quarter of a sphere. As a consequence, a great portion of contacts happening on the regions outside the grasp closure is undetectable. In Padmanabha et al. (2020), this issue is mitigated by the use of five endoscope micro cameras looking at different regions of the finger. However, this results in a significant increase in cost for the sensor, according to the authors, approximately US$3200 (vs. only around US$100 for ours).

## 3. The GelTip Sensor

### 3.1. Overview

**Figure 2:**
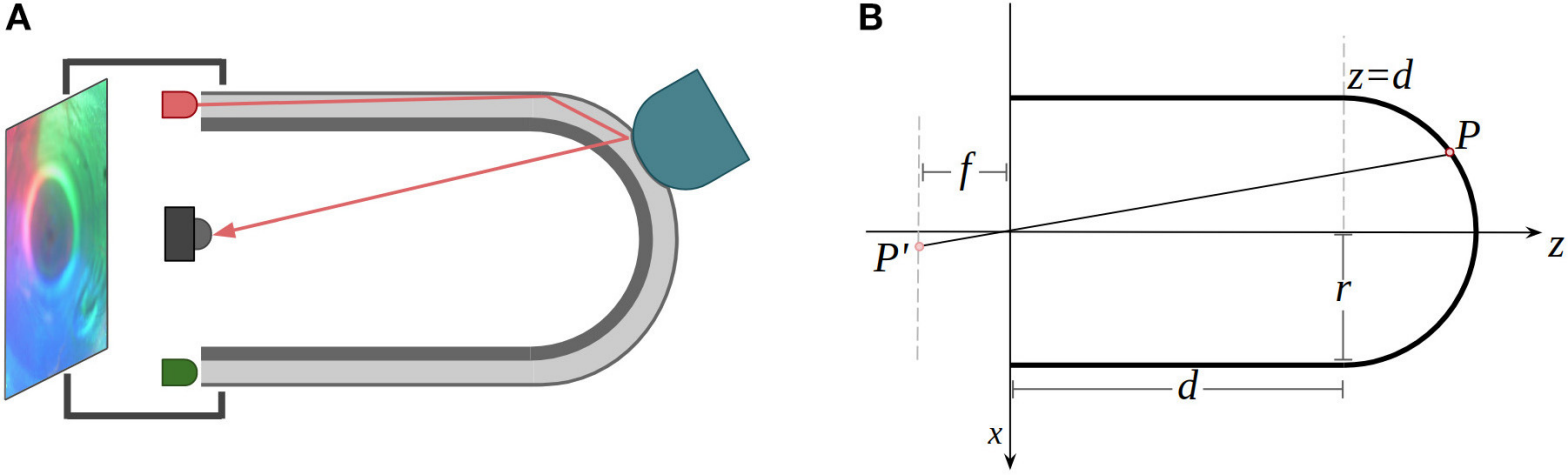
**(A)** The working principle of the proposed *GelTip* sensor. The three-layer tactile membrane (rigid body, elastomer, and paint coating) is shown in gray. The light rays emitted by the LEDs travel through the elastomer. As one object, shown in yellow, presses the soft elastomer against the rigid body, an imprint is generated. The resulting image is captured by the camera sensor, placed in the core of the tactile sensor. An opaque shell, enclosing all the optical components, ensures the constant internal lighting of the elastomer surface. **(B)** Two-dimensional representation of the geometrical model of the *GelTip* sensor. The tactile membrane is modeled as a cylindrical surface and a semi-sphere. An optical sensor of a focal-length *f* is placed at the referential origin of the sensor, which projects a point on the surface of the sensor *P* into a point *P*′ in the image plane. The sensor has a radius *r*, and its cylindrical body has a length *d*.

As illustrated in Figure 2A, the introduced GelTip optical tactile sensor has a fingertip shape. The sensor body consists of three layers, from the inside to the outer surface: a rigid transparent body, a soft transparent membrane, and a layer of opaque elastic paint. A camera is placed at the base of the sensor, looking from the inside of the tube. When an object is pressed against the tactile membrane, the elastomer distorts and indents the object shape. The camera captures the obtained imprint into a digital image. Since one property of tactile sensing is being immune to external light variations, the camera is enclosed within an opaque shell, with the tactile membrane being the only interface with the external environment. Thanks to the finger shape of the sensor, the LED light sources can be placed adjacent to the base of the sensor and the strategically controlled light rays are guided through the tube and elastomer.

### 3.2. The Sensor Projective Model

The tactile images captured by the sensor can be processed to retrieve information about the contacted surfaces, that is, the geometry of the object, the contact location, force distributions, and so on. Methods for obtaining such information have been introduced in previous works (Li et al., 2014; Yuan et al., 2017). However, due to having a flat surface for the tactile membrane, the relationship between the camera and the elastomer surfaces has not been explicitly considered in all of these works of previous GelSight sensors. In this subsection, we derive the protective function *m* that maps pixels in the image space (*x*′, *y*′) into points (*x, y, z*) on the sensor surface. The camera is assumed to be placed at the referential origin, looking in the direction of *z* axis. The sensor space takes the center of its base, which is also the center point of the camera, as the coordination origin (0, 0, 0); the image space takes the center of the image as the origin (0, 0). Such a projection model is necessary for, among other applications, detecting the position of contacts in the 3D sensor surface.

As illustrated in Figure 2B, the sensor surface can be modeled as a joint semi-sphere and an opened cylinder, both sharing the same radius *r*. The cylinder surface center axis and the *z*-axis are collinear, therefore, the center point of the semi-sphere can be set to (0, 0, *d*), where *d* is the distance from the center point of the base of the semi-sphere to the center point of the base of the sensor. The location of any point on the sensor surface (*x, y, z*) can be represented as follows:

$$\begin{array}{l} \{\begin{array}{ll} x^{2}+y^{2}+{\left(z-d\right)}^{2}=r^{2} & \text{for }z>d\;\left(1\right)\\ x^{2}+y^{2}=r^{2} & \text{for }z<=d\;\left(2\right) \end{array} \end{array}$$

By making the usual thin lens assumptions, we model the optical sensor as an ideal pinhole camera. The projective transformation that maps a point in the world space *P* into a point in the tactile image *P*′ can be defined using the general camera model (Szeliski, 2010) as:

(3) $$\begin{array}{ll} P^{\prime } & =K\left[R|t\right]P \end{array}$$

(4) $$\begin{array}{l} K\;=\left[\begin{array}{c} \begin{array}{llll} fk & 0 & c_{x} & 0\\ 0 & fl & c_{y} & 0\\ 0 & 0 & 1 & 0 \end{array} \end{array}\right] \end{array}$$

where *P*′ = [*x*′*z, y*′*z, z*]*^T^* is an image pixel and *P* = [*x, y, z*, 1]^*T*^ is a point in space, both represented in homogeneous coordinates here, [*R*|*t*] is the camera's extrinsic matrix that encodes the rotation *R* and translation *t* of the camera, *K* is the camera intrinsic matrix (*f* is the focal length; *k* and *l* are the pixel-to-meters ratios; *c*_*x*_ and *c*_*y*_ are the offsets in the image frame). Assuming that the used camera produces square pixels, that is, *k* = *l*, *fk* and *fl* can be replaced by α, for mathematical convenience.

The orthogonal projections in the *XZ* and *YZ* of a generic projection ray can be obtained by expanding the matrix multiplication given by Equation (3) and solving it with respect to *x* and *y*.

(5) $$\begin{array}{l} \{\begin{array}{l} x^{\prime }z=\alpha x+c_{x}z\\ y^{\prime }z=\alpha y+c_{y}z\Leftrightarrow \{\begin{array}{c} \alpha x=x^{\prime }z-c_{x}z\\ \alpha y=y^{\prime }z-c_{y}z \end{array}\Leftrightarrow \{\begin{array}{c} x=(\frac{x^{\prime }-c_{x}}{\alpha })z\\ y=(\frac{y^{\prime }-c_{y}}{\alpha })z \end{array}\\ \;z=z \end{array} \end{array}$$

The desired mapping function *m*:(*x*′, *y*′) → (*x, y, z*) can then be obtained by constraining the *z* coordinate through the intersection of the generic projection ray with the sensor surface, described in Equation (6). The discontinuity region, that is, a circumference, is found by setting *z* = *d* in Equation (5). The complete derivation of *z* can be found in the Supplementary Material.

$$\begin{array}{l} m(x^{\prime },y^{\prime })=\left\{ \begin{array}{l} x=(\frac{x^{\prime }-c_{x}}{\alpha })z\\ y=(\frac{y^{\prime }-c_{y}}{\alpha })z\\ z=\{\begin{array}{ll} \sqrt{\frac{{\left(r\alpha \right)}^{2}}{{\left(x^{\prime }-c_{x}\right)}^{2}+{\left(y^{\prime }-c_{y}\right)}^{2}}} & \text{if }{\left(x^{\prime }-c_{x}\right)}^{2}+{\left(y^{\prime }-c_{y}\right)}^{2}<{\left(\frac{r\alpha }{d}\right)}^{2}\\ \; & \;\\ \frac{\alpha^{2}2d+\sqrt{{\left[-\alpha^{2}2d\right]}^{2}-4[{\left(x^{\prime }-c_{x}\right)}^{2}+{\left(y^{\prime }-c_{y}\right)}^{2}][(d^{2}-r^{2})\alpha^{2}]}}{2[{\left(x^{\prime }-c_{x}\right)}^{2}+{\left(y^{\prime }-c_{y}\right)}^{2}+\alpha^{2}]} & otherwise \end{array} \end{array}\right. \end{array}$$

**Figure 3:**
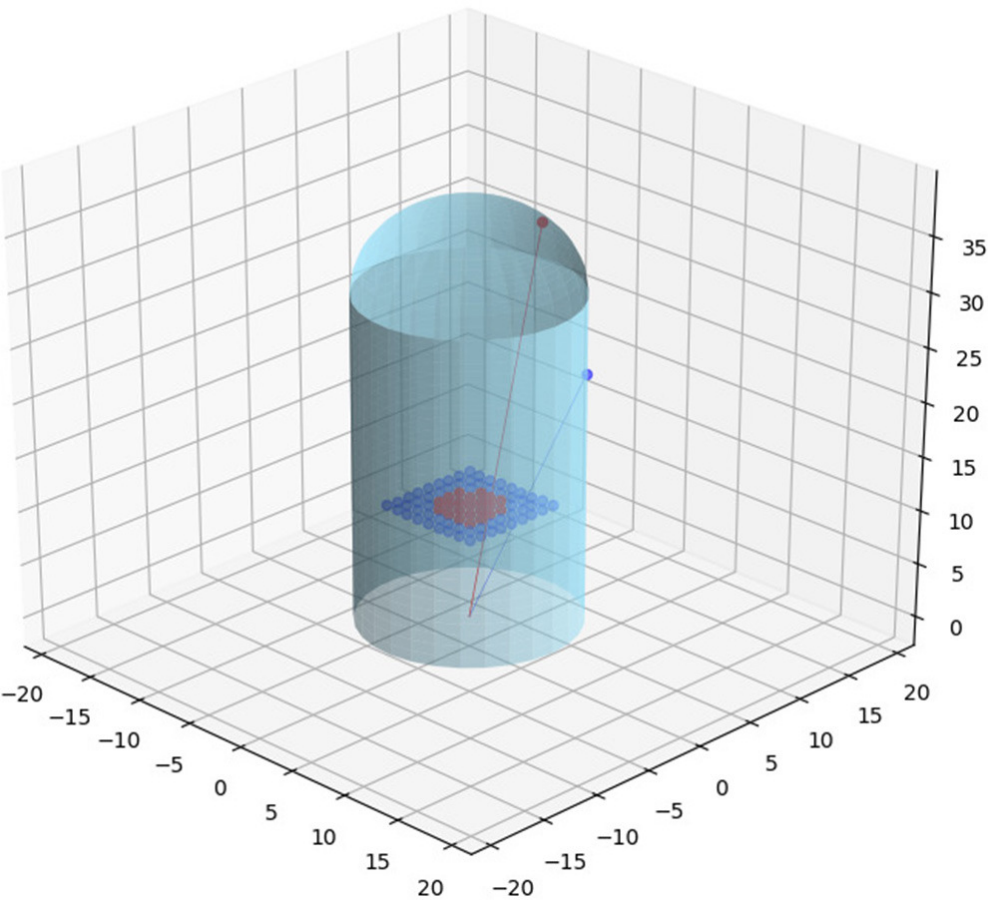
Two projection rays that correspond to the spherical (in red) and cylindrical (in navy blue) regions are depicted in the figure. Each ray intersects three relevant points: the frame of reference origin, a point in the sensor surface, and the corresponding projected point in the image plane.

The introduced sensor model is validated and visualized in Figure 3. Two projection rays, corresponding to the spherical and cylindrical regions, are depicted. Each ray intersects three relevant points: the frame of reference origin, the point in the 3D sensor surface, and the corresponding projected point in the image plane.

### 3.3. Fabrication Process

With its fingertip-shaped design, the *GelTip* sensor can be mounted onto robotic grippers and serve as a replacement for existing senseless fingers/fingertips. Due to its compact size and the non-flat surface, the major challenge to address in the development of the GelTip sensor is the fabrication of the tactile membrane. On the other side, because of the finger-like shape, the elastomer can grab better onto the non-flat body and be more robust to external tangential forces. In addition, the bent elastomer can be used to guide the light rays and minimize the necessary illumination apparatus, compared to the GelSight sensor of a flat membrane (Li et al., 2014).

An off-the-shelf transparent test tube is used to construct the rigid layer of the sensor body, simplifying (or avoiding) the fabrication of the necessary curved surface. The commercially available tubes are made up of plastic/acrylic or glass, and are sold for experimental or decorative purposes. While initially we experimented with glass tubes, we quickly found that trimming a tube made up of such material is far too complicated. Because of that, we turned to plastic/acrylic tubes. One disadvantage of using the off-the-shelf test tubes, particularly the plastic ones, is that they contain small imperfections resulted from the manufacturing process. An example is the discontinuity between the semi-spherical and cylindrical regions. A second example is that an imprint is often found at the center of the semi-spherical region of the test tube. An alternative approach would be to print the rigid tube using a stereolithography 3D-printer and clear resin; however, proper polishing would be necessary to ensure its optical transparency. For this reason, we did not explore this approach in this work.

**Figure 4:**
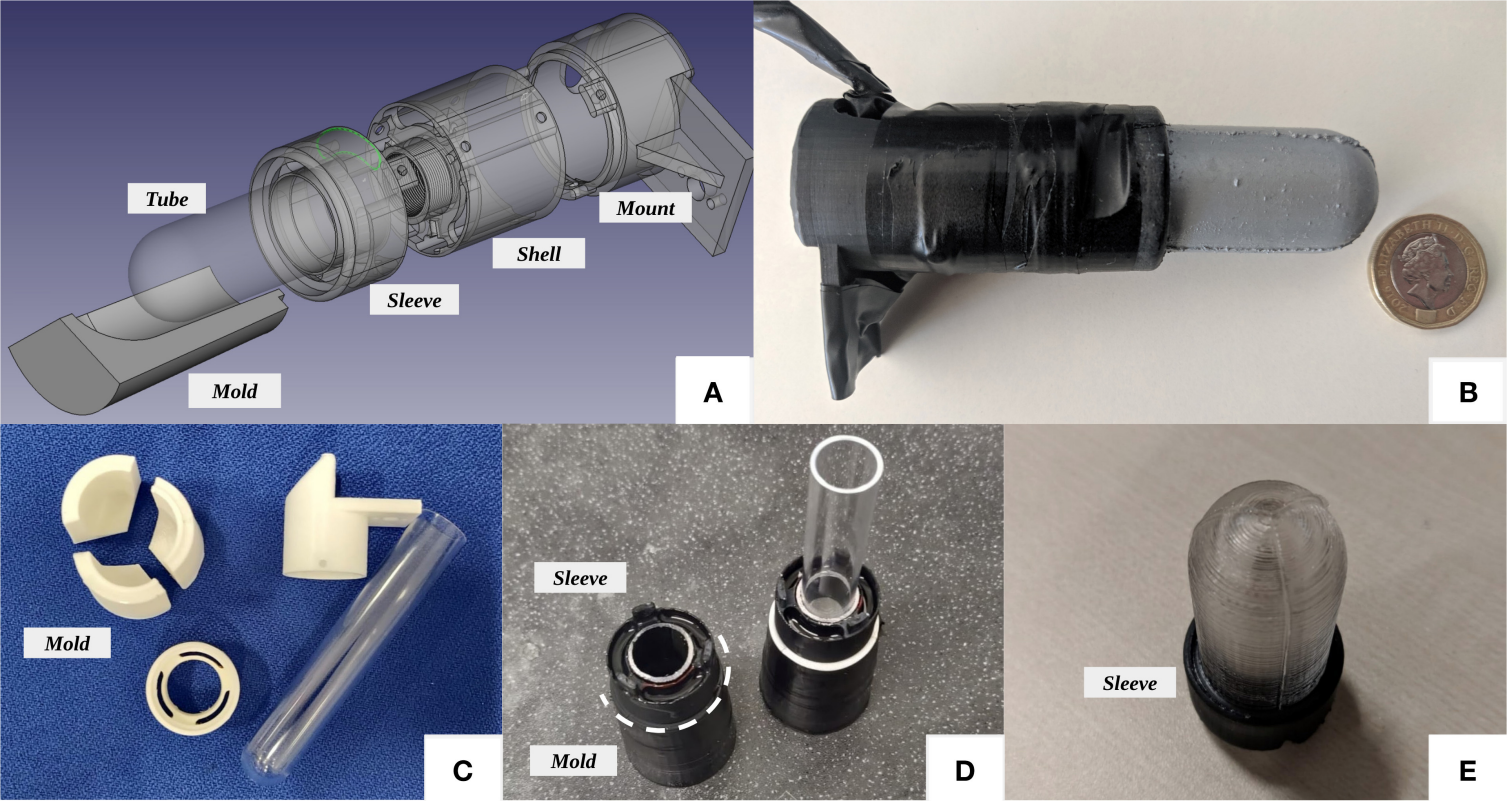
**(A)** Exploded view of the GelTip tactile sensor design. **(B)** A GelTip sensor, next to a British one pound coin, for relative size comparison. The sensor has a length of approximately 10 cm, its shell has a diameter of 2.8cm, and the tactile membrane has a length of 4cm and a diameter of 2cm. **(C)** The three-part *mold* next to the remaining parts used in the GelTip construction. **(D)** The plastic tube is inserted into the sleeve and then mounted onto the mold, afterwards the tube is measured and trimmed and then the elastomer is poured. **(E)** The tactile membrane after being de-molded and before being painted.

As shown in Figure 4A, the remaining necessary rigid parts to build the sensor body are a *shell*, where the camera electronics and LEDs are installed, a *sleeve* that is glued onto the test tube and tightened to the *shell*, and a *supporting base* that is used to bolt the sensor into the fingers of the robot gripper and host the main electronics. Furthermore, a three-part *mold* is used to fabricate the elastomer with the desired thickness and shape. To fabricate the rigid parts, we take advantage of 3D printing technology. We experiment with printing the parts using both fused filament fabrication (FFF) and stereolithography (SLA) printers, that is, the Anycubic i3 Mega and the Formlabs Form 2. The 3D-printed parts are shown in Figure 4C. The models and further information about the GelTip sensor are available at http://danfergo.github.io/geltip.

Having the mold and test tube ready, as shown in Figure 4D, that is, the test tube is inserted into the sleeve and mounted onto the mold; afterwards the tube is measured and trimmed. The silicone for the soft membrane is then poured through the LED slits. Because of the three-part design, once the elastomer is cured, the mold can be opened from the bottom to reveal the tactile membrane, as shown in Figure 4E. We use the same materials as suggested in Yuan et al. (2017), that is, XP-565 from Silicones, Inc. (High Point, NC, USA) and Slacker® from Smooth-on Company. After extensive experiments, we find the best ratios to be 1:22:22, that is, we mix 1 g of XP-565 part-A, 22 g of XP-565 part-B, and 22 g of the Slacker. This amount of mixture is sufficient to fabricate two sensor membranes. The ratio *part-A/part-B* influences the rigidity of the elastomer, that is, higher concentration of *part-B* produces a softer silicone. The Slacker, on the other hand, contributes to the silicone tackiness. It is necessary to add sufficient Slacker to make the elastomer be able to capture high-frequency imprints such as a fingerprint. However, it will make the silicone sticky if too much slacker is added.

After the elastomer is cured and de-molded, we proceed with painting. The off-the-shelf spray paints tend to form a rigid coat and cracks will develop in the coat when the elastomer deforms or stretches. To avoid these issues, we fabricate a custom paint coat using the airbrush method suggested in Yuan et al. (2017). We mix the coating pigment with a small portion of *part-A* and *part-B* of XP-565, with the same ratio used in the elastomer. We experiment with both the Silver Cast Magic® from the Smooth-on Company and the aluminum powder (1 μm) from the US Research Nanomaterials, Inc. After mixing them properly, we dissolve the mixture using a silicone solvent until we achieve a watery liquid. The liquid paint is then sprayed onto the elastomer surface using an airbrush. It is essential to apply the paint using low pressure and at a sufficient distance, and have the surface rest between coats, so that a smooth surface finish can be achieved. We use a ratio of 1:20:5 for *part-A, part-B* and the pigment powder, respectively.

Three sets of LEDs are then soldered, either of different colors, red, green, and blue, or all white. They are inserted into three corresponding pockets in the sensor *sleeve*. Since different LEDs emit different light intensities, we solder each cluster to a different wire and resistor before connecting them to the power source. The values of these resistors are manually tuned and vary from 30 to 600 Ω. The power source can be either extracted from the camera USB cable, by splicing it, or adding a secondary USB cable.

At the core of this sensor lies a Microsoft LifeCam camera (either the Cinema or Studio version). We choose these cameras because of the fact that their main circuit board is orthogonal to the optical sensor. This configuration enables us to use it within the compact cylindrical finger shell. By default, the cameras are equipped with a viewing angle 73 ° lens. To have a larger Field of View (FoV), we replace the lens by a 170 ° wide angle M12 lens.

The final prototype can be seen in Figure 4B. The sensor, placed next to a British 1 pound coin, has a length of approximately 10 cm; its shell has a diameter of 2.8 cm; and the tactile membrane has a length of 4 cm and a diameter of 2 cm. In **Figure 11**, example contacts with human fingerprints and an open-cylinder solid are also provided for qualitative evaluation. In Table 2, we provide a summary of the steps of the process and their corresponding durations for fabricating a GelTip sensor.

**Table 2:** Manufacturing steps and their corresponding approximate durations.

**Steps**	**Durations**
Sensor parts and molds printing	3 h *FFF printing*
	4.5 h *SLA printing* + 1 h *washing* + 1 h *curing*
Elastomer preparation and pouring	*mixing* + *cut&glue tube* + 1 h *vacuum degassing*
Elastomer curing	2+ days *in the ambient temperature*
Electronics	1 h *camera dissassembly* and *LEDs soldering*
Painting	1 h *paint fabrication* and *application*
Paint drying	2+ days *in the ambient temperature*
Final assembly	15 min *for assembly and focal-length adjustment*

*It should take about 1 week to make a GelTip sensor by hand; however, the durations for the elastomer to cure and for the paint to dry depend significantly on the ambient conditions (temperature, etc.) of the manufacturing site*.

## 4. Evaluation

In this section, we summarize the results of our experiments. In the first group, a set of parameters of the sensor, that is, the test tube radius, the viewing angle of the camera lens, the painting and illumination, and the surface roughness, are investigated. Then we demonstrate that the GelTip sensor can effectively localize the contacts at different locations of the finger body, with a small localization error. Finally, the advantages of leveraging all-around touch sensors in the context of manipulation tasks, together with other remote sensors, are evaluated by executing a set of grasp experiments in a Blocks World environment.

### 4.1. Sensor Construction Parameters

In section 3, the GelTip generic model and fabrication process have been described. In this section, we summarize the results obtained during experiments carried to determine the optimal settings for parameters that highly affect the quality of the obtained tactile image.

#### 4.1.1. The Radius of the Sensor Test Tube

**Figure 5:**
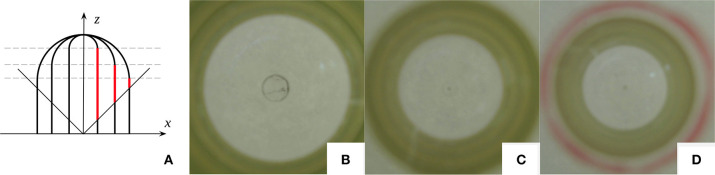
**(A)** Three sensor designs of different radiuses and their lateral observable areas (highlighted in red). It should be noted that for tubes with the same length (given the same camera viewing angle), the larger the radius is, the shorter is the lateral observable area. **(B–D)** Images captured using three test tubes of different diameters: 21, 15, and 13mm, respectively. The three have the same depth *d* of 3cm. A yellow and green electrical tape is placed on the cylindrical region of the tube, tangent to the discontinuity line. The tape has a width of approximately 14.5mm, and each of its green stripes has a width comprised between 1 and 2mm. For the thinner tube, an additional red line is traced approximately 2mm from the tape. The same camera and wide lens are used to capture the images.

One key parameter to consider when designing the GelTip sensor is its tactile membrane radius. Given a fixed length, the radius influences not only the sensor compactness but its lateral observable area as well, as depicted in Figure 5A. Samples showing the practical consequence of this effect are shown in Figures 5B–D, where three images are captured using three test tubes of different inner diameters, that is, 21 , 15 , and 13 mm, with the same depth *d* of 3 cm. A yellow and green electrical tape is placed on the cylindrical region of the tube, tangent to the discontinuity line. The tape width is approximately 14.5 mm and each of its green stripes width is between 1 and 2 mm. For the thinner tube, an additional red line is also traced, approximately 2 mm from the tape. The same camera and wide lens are used to capture the images. As seen in the figures, the larger the tube, the further away the optical rays intersect it, and consequently the smaller is the observable side area. As we aim to develop a sensor that is capable of perceiving the maximum side area, we use the tube with an inner diameter of 15 mm, which is the minimal radius to fit our M12 lens inside. This configuration offers an intermediate compromise between observable side areas, sensor compactness, and flexibility for adjusting the positioning of the camera sensor.

#### 4.1.2. The Viewing Angle of the Camera Lens

Similar to the finger radius, the camera viewing angle is another parameter that highly affects the size of the observable area, as demonstrated in Figure 5. The shown pictures capture the internal views of the sensor, using lenses of two different viewing angles: 70 ° and 170 ° respectively. In this experiment, a test tube with an inner diameter of 15 mm is used. When a standard 70 ° lens is used, only the area of the tip of the finger is visible, while with a wider lens the side area is also visible. To this end, the camera lens with a viewing angle 170 ° is selected, so that the GelTip sensor is capable of sensing contacts on both its tip and sides.

#### 4.1.3. Painting and Illumination

**Figure 6:**
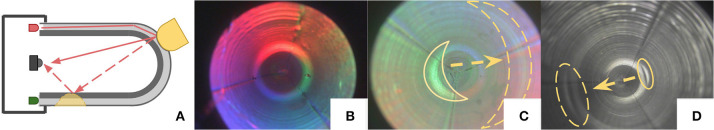
**(A)** Illustration depicting the cause of *ghost contacts*. Due to the sensor geometry, lighting, and surface characteristics, the light rays initially guided through the elastomer are diverged by a (real) contact imprint, and consequently are projected onto the opposite side of the sensor, resulting in a second *ghost contact*. **(B–D)** Comparison of different paints: silicone with aluminum powder (only), metallic elastic paint and silicone with a mix of aluminum and Silver Cast Magic® powders. In **(B)** and **(C)**, RGB lighting is used, and in **(D)** white lighting is used. In **(B)** and **(C)**, due to the illumination and paint reflectiveness, the discontinuity region generates a bright reprojection on the opposite side of the sensor. In **(D)**, the contact existing in that region is also projected as an artifact that we refer to as *ghost contact*.

In Yuan et al. (2017), aluminum powder paint is suggested to reduce the existence of specular reflections, so as to improve the image quality for surface reconstruction. In our experiments, we find that due to the curved surface of the GelTip, this powder ends up absorbing most of the light that results in a highly non-uniform color distribution. Furthermore, the absorption of the light makes the tip of the finger to be poorly illuminated. As a consequence, there exists a darker region at the tip and a poor tactile image is observed when contacts are applied to the tip. As depicted in Figure 6, we compare the views of two tactile membranes painted differently by the aluminum powder and the metallic elastic paint. To obtain homogeneous light distribution, we mix Silver Cast Magic® powder into the aluminum powder to increase the reflectiveness of the coating. It should be pointed that the chance of having *ghost contacts*, that is, internal reprojections that resemble real contacts, increases when the paint reflectiveness and light brightness are excessive. The effect of the *ghost contact* is illustrated in Figure 6A and an example of such *ghost contacts* can be seen in Figure 6D, where a contact happening near the sensor discontinuity region is projected onto the opposite side of the sensor. A larger but less prominent reprojection, in this case caused solely by the sensor self-curvature, is also seen in most samples captured with tactile membranes coated with the semi-specular paints, as highlighted in Figure 6C.

#### 4.1.4. Surface Roughness

**Figure 7:**
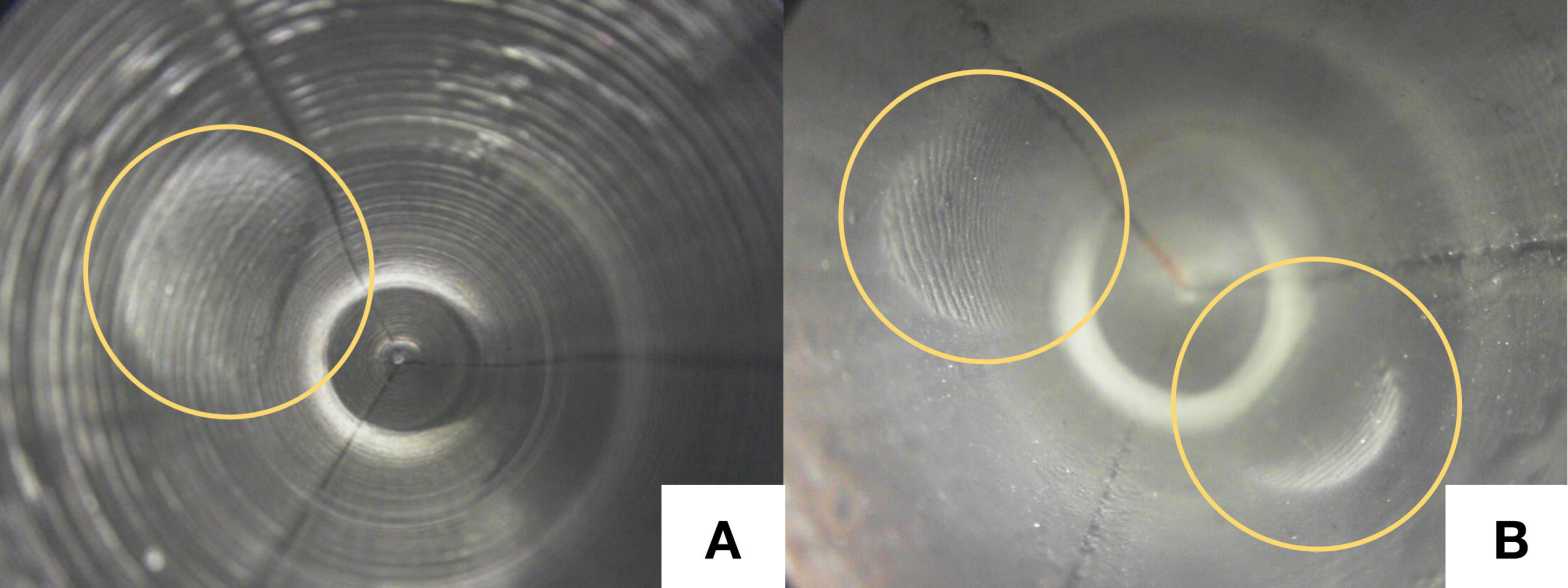
Elastomer textures obtained using **(A)** fused filament fabrication (FFF) and **(B)** stereolithography (SLA) 3D printers. As highlighted in **(B)**, the ridges of human fingerprints are camouflaged by the elastomer ridges.

In Donlon et al. (2018), the usage of textured fabric is discussed as an approach to increase the responsiveness of the tactile signal. We experiment with printing the necessary molds for shaping the elastomer using both the Anycubic i3 mega (FFF) and the Formlabs Form 2 (SLA) 3D printers. Consequently, two differently textured elastomers are obtained, as shown in Figure 7. The FFF printing process results in a textured surface with ridges that run parallel to the sensor base, while with the SLA printer a smoother surface is obtained. Specks that result from the currently imperfect painting process are also visible on both surfaces. As seen in the figure, ridges in the elastomer make it more difficult to identify high-frequency textures such as human fingerprints. From handling the sensor, we can also point that the textured elastomer has a lower friction coefficient than the non-textured elastomer.

### 4.2. Contact Localization

**Figure 8:**
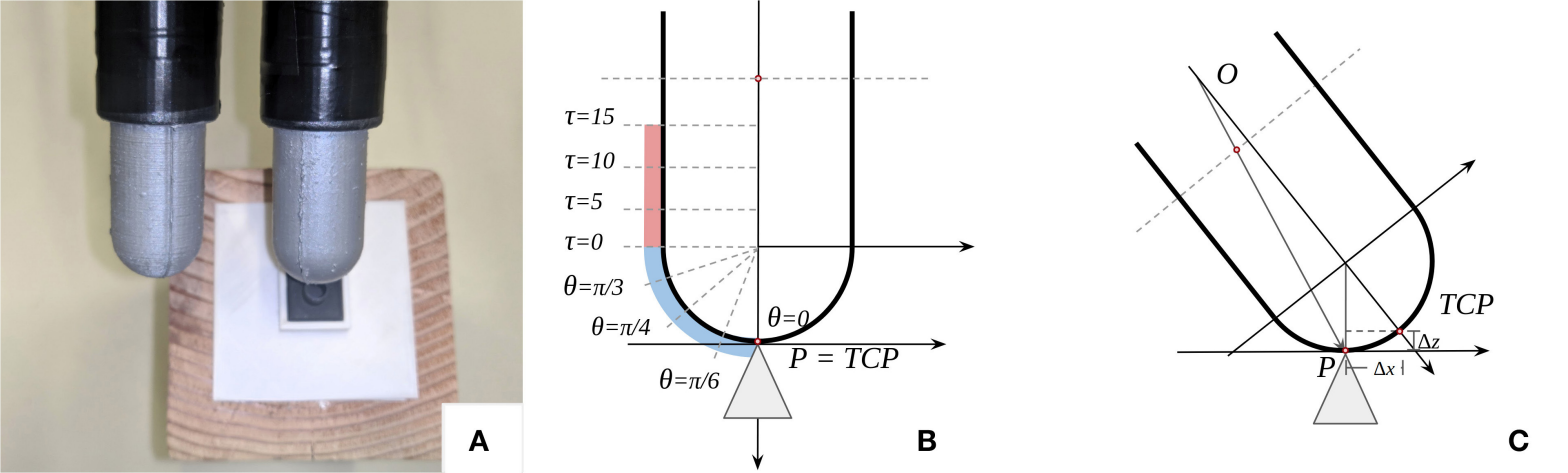
**(A)** Two GelTip sensors are installed on a robotic actuator and a 3D-printed mount that holds a small 3D-printed shape (a cylinder here) placed on top of a wooden block. The actuator moves in small increments and collects tactile images annotated with the known contact positions. **(B,C)** Illustration of the motion of the sensor during the data collection. The sensor starts pointing downwards, as shown in **(B)**. To obtain contacts on the sensor surface, while moving, the sensor is also translated by (Δ*x*, Δ*z*), as shown in **(C)**. A total of eight contacts are collected per object: four rotations (θ) on the sensor tip and four translations (τ) on the sensor side, as highlighted in **(B)**.

As an optical tactile sensor of a finger shape, the main feature of the GelTip sensor is the detection of contacts throughout the entire surface of the finger. To evaluate this capability, we carry out a contact localization experiment, in which contacts at the sensor tip and sides are measured. To this end, the GelTip sensor is installed on a robotic actuator that rotates and translates to tap objects at multiple known positions of its finger surface, as illustrated in Figure 8.

#### 4.2.1. Experiment Setup

A set of small objects is 3D printed using the Formlabs Form 2 SLA 3D Printer. The set contains seven different objects: a cone, a sphere, an irregular prism, a cylinder, an edge, a tube, and a slab, as shown in Table 3. Each object has a maximum dimension of 1 × 1 × 2 cm^3^. A 3D-printed mount is also built to ensure that all the objects are kept in the same position throughout the experiment. The GelTip sensor is installed on a robotic actuator, that is, the 6-DOF Universal Robots UR5 arm with a Robotiq 2F-85 gripper. The 3D-printed mount is placed on top of a raised surface. For the sake of inverse kinematics, the actuator Tool Center Point (TCP) is set as the sensor tip.

**Table 3:** Summary of the contact errors expressed in millimeters.

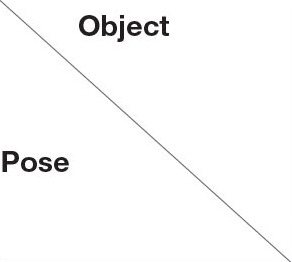		** 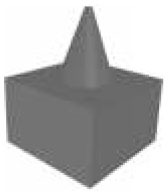 **	** 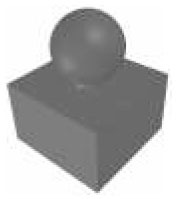 **	** 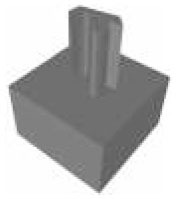 **	** 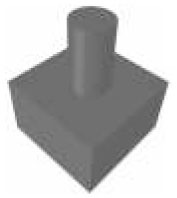 **	** 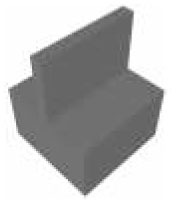 **	** 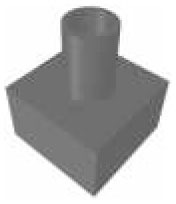 **	** 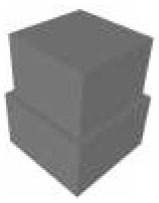 **	
		**Cone**	**Sphere**	**Irregular**	**Cylinder**	**Edge**	**Tube**	**Slab**	**$\bar{x}\pm \text{s}$**
**Rotations (θ)**
	0	3.81	4.77	4.81	4.85	3.88	6.19	4.17	4.17 ± 0.75
	π/6	0.36	1.70	1.68	2.45	1.63	2.88	3.33	2.01 ± 0.90
	π/4	0.79	1.00	0.86	0.45	1.61	0.69	1.81	1.04 ± 0.46
	π/3	5.85	9.95	8.05	2.34	16.79	2.74	3.01	6.96 ± 4.82
**Translations (τ)**
	0	9.99	12.73	11.94	2.41	14.00	1.96	2.06	7.87 ± 5.08
	5	4.81	10.69	7.76	7.90	9.37	6.05	9.66	8.03 ± 1.92
	10	2.55	13.02	8.52	13.67	11.73	3.24	0.74	7.55 ± 5.00
	15	0.85	0.43	1.28	2.48	0.76	2.91	25.35	4.86 ± 8.41
$\bar{x}\pm s$		3.63 ± 3.26	6.79 ± 5.38	5.61 ± 4.08	4.57 ± 4.30	7.47 ± 6.29	3.33 ± 1.90	6.27 ± 8.17	5.38 ± 5.07

*Contacts are localized in the image space, using the contact localization algorithm (see section 4.2.2). The detected contact is then projected into the world coordinates, using the sensor projective model (see section 3.2). Finally, the contact error is calculated as the Euclidean distance between the detected and true contact points*.

The actuator starts with its fingers pointing downwards, that is, orientation 0. The actuator is then visually aligned using the cone object. Contacts are then registered, first on the sensor tip, by rotating the sensor, and then on the side, by translating the sensor. In Figure 5B, markings show the location of such contacts, and in Figure 5C, the necessary (Δ*x*, Δ*z*) translation to obtain contacts on the finger skin is also shown.

#### 4.2.2. Contact Localization Algorithm

To automatically detect the positions of such contacts, a simple image subtraction-based algorithm is implemented. Before each contact, a reference image is captured. When a contact is applied, the element-wise absolute difference between the reference and the in-contact frames is computed. The obtained difference frame is filtered with per-channel 15 × 15 2D mean convolutions, which are then averaged and normalized channel-wise, to obtain a heat map of the contact regions. Pixels with a likelihood lower than 60% are discarded, that is, set to zero. In the next step, the OpenCV *findContours* operation is used to extract cluster regions. Such clusters are further filtered based on the size of their area, that is, only clusters with an area between 0.012 and 0.04% of the picture area are kept. The OpenCV *fitEllipse* function is then applied to each cluster to find their center points. To mitigate the potential effect of outliers, and since in our experiments we are interested in predicting a single contact per frame, a final prediction is set as the weighted average of the cluster centers. The sensor projection model (see section 3) is then used to get the contact position on the sensor.

#### 4.2.3. Projection Model Calibration

To use the projection model described in section 3, five parameters are necessary to be known: *r*, *d*, *c*_*x*_, *c*_*y*_, and α. The first two are extracted from the dimensions of the sensor design; however, the latter three are the intrinsic parameters of the camera, which need to be calibrated. To this end, we obtain such parameters from a known pair of corresponding (*x*′, *y*′) and (*x, y, z*) points. We set the actuator to tap the object in the 15 mm translation position. The center of the sensor tip (*c*_*x*_, *c*_*y*_) and the contacted point are manually annotated in the image space. The α parameter can then be derived by fitting the known information into Equation (5).

#### 4.2.4. Results of the Contact Localization

After detecting the contact in the image space and projecting it into (*x, y, z*) coordinates, the Euclidean distance between the predicted and the true contact positions is computed. For each of the seven objects, a total of eight contacts are recorded, that is, four rotations (θ): 0, π/6, π/4, π/3; and four translations (τ): 0, 5, 10, 15mm. The resulting localization errors, expressed in millimeters, are summarized in Table 3. Overall, the variance between the observed localization errors is substantial; in some contacts the obtained errors are lower than 1 mm, while in others are over 1 cm. On the other hand, the localization error, for each object or position, is correlated with its variance. The largest localization errors occur on objects with large or rounded tops, that is, sphere, edge and slab; contrariwise, the lowest errors are observed for objects with sharp tops, that is, cone, tube and cylinder. In terms of the localization errors at different positions, contacts occurring near the sensor tip, that is, the rotations, present lower errors than contacts occurring on the sensor side, that is, translations. In particular, contacts occurring at *pi*/4 and *pi*/6 have the lowest errors.

**Figure 9:**
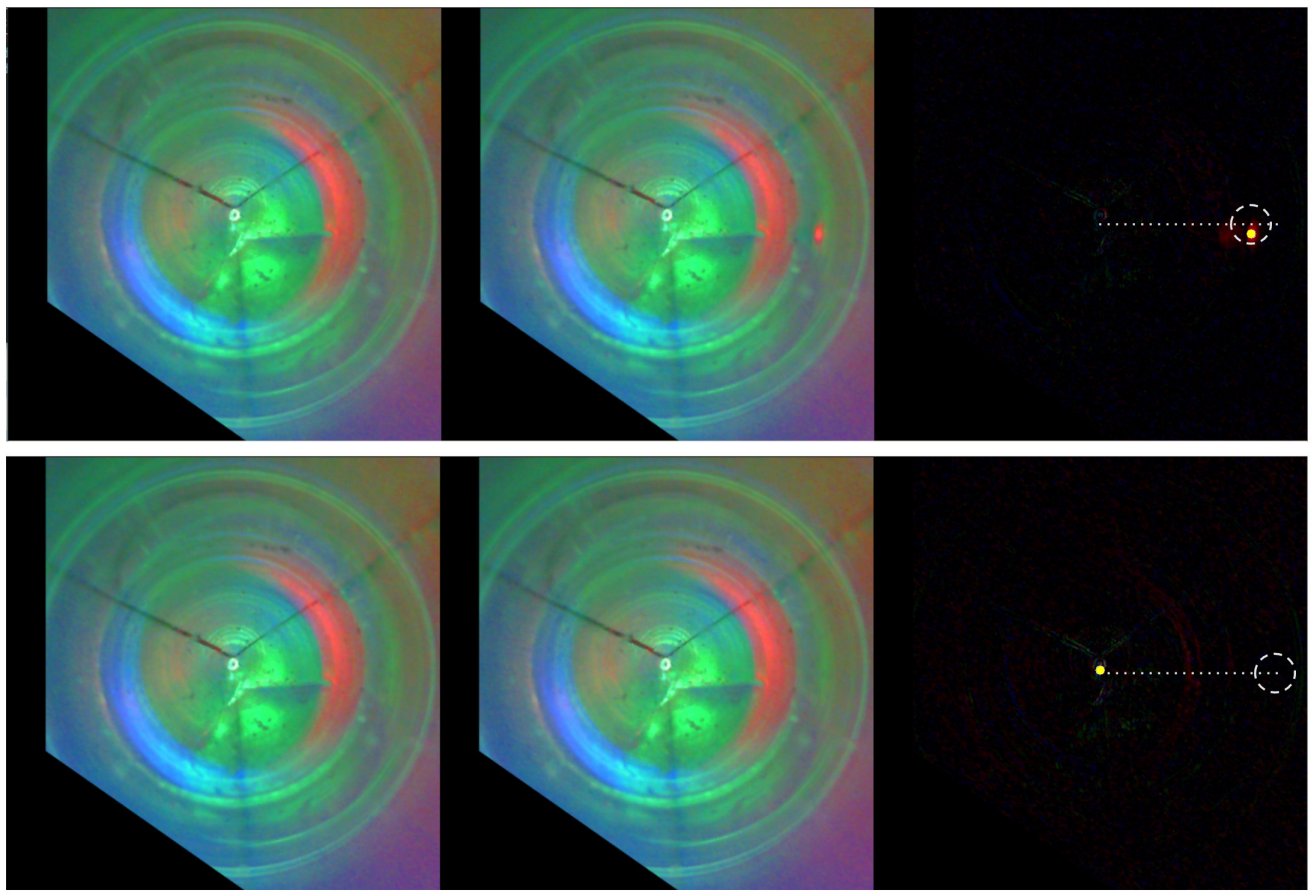
Contact localization for the smallest and largest errors, that is, < *Cone*, θ = 0 > and < *Slab*, τ = 15*mm* >. From left to right: the reference frame, the in-contact frame, the computed pixel-wise absolute difference. The contact region (dash circumference), the predicted contact position (yellow circle), and the axis where the contacts occur (dotted line) are highlighted in the right most frame.

From the data and our observations during the experiment, we conclude that the obtained errors arise from three sources: (1) week imprints, (2) the flexing of the sensor, and (3) imperfections in the sensor modeling and calibration. Examples of captured tactile images and corresponding predictions are for the smallest (i.e., < *Cone*, θ = 0>) and largest localization errors (i.e., < *Slab*, τ = 15 mm>), as shown in Figure 9. In the first case, due to the bright imprint provided by the sharp cone top, the algorithm successfully locates the contact. In the second case, due to the imperceptible contact imprint, the algorithm incorrectly predicts the contact in the sensor tip.

### 4.3. Touch-Guided Grasping in a Blocks World Environment

**Figure 10:**
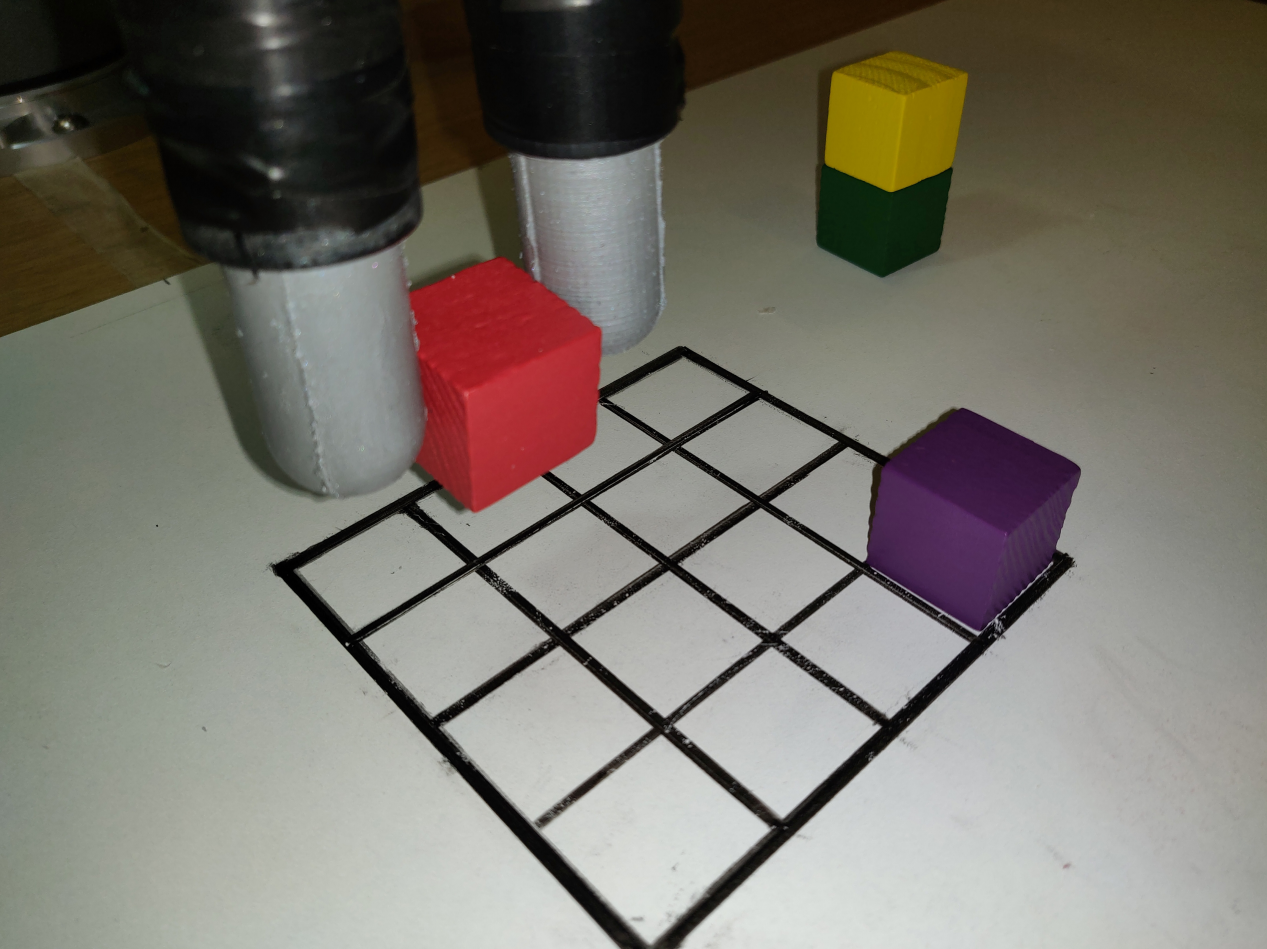
The Blocks World experimental setup. In each experiment, the robot actuator moves row by row, attempting to grasp each block. The experiment shows that, even with the initial uncertainty, the robot grasps all the blocks successfully using the all-around touch feedback.

In the task of grasping objects, the initial motion of the gripper is often planned using remote sensing, for example, camera vision and Lidar. However, remote sensing suffers from occlusions and inaccurate predictions about geometry and distances. In such cases, the final grasp and re-grasp control policies have to rely on inaccurate information of where and when contacts occur. In contrast, touch sensing offers accurate feedback about such contacts. To demonstrate how the all-around sensing of the GelTip sensor will facilitate such situations, we conduct a set of experiments of grasping objects in a Blocks World environment, as shown in Figure 10.

#### 4.3.1. Experiment Setup

To mimic the inaccuracies from remote sensing (in its worst extreme), in this experiment, each grasp attempt is randomly selected, and therefore no camera or any other visual system is used. As such, the hardware setup consists of two GelTip sensors installed on a Robotiq 2F-85 gripper that is mounted onto a 6-DOF Universal Robot UR5 robot arm. Wooden blocks from the YCB object set (Calli et al., 2015) are randomly placed on a 4x4 board, positioned under the gripper. Each block has a side of 2.5 cm and only one block is placed in each row. The robot attempts to grasp each block and remove it from the board, with the goal of clearing the board. The robot starts at one row and moves to the opposite iteratively. While approaching an object, if a collision happens against the sensors tips, that is, outside the grasp closure, the motion is halted. If the arm reaches a minimal known height, the gripper is closed. While closing, if a contact is detected inside its grasp closure, it means that an object is being grasped and the gripper is stopped. Otherwise, it is considered an unsuccessful attempt. After five unsuccessful attempts of grasping the same block, the robot skips to the next one. Blocks left on the board at the end of a given run are considered as failures.

#### 4.3.2. Compared Policies

Three policies compared are as follows: (1) ***Random grasp***(Rg) that mimics the inaccuracies from remote sensing–based grasping by sampling the block position, that is, column, from a uniform random distribution [0, 3]. (2) ***Random grasp + Touch informed regrasp***(RgTr). It chooses the grasp position in the same manner as **Random grasp**. However, when a collision occurs during the grasping motion, it exploits the contact information to perform a regrasp by moving toward the column in which the contact was detected. (3) ***Controlled policy***(C) that is implemented as a reference. In this case, the agent always knows the position of each block and consequently always moves directly toward it.

#### 4.3.3. Results of the Grasping Experiment

For each of the three policies, each run is repeated five times, and the obtained results are summarized in Table 4. In all the measured metrics, RgTr is a more successful policy than Rg. For instance, Rg fails to grasp 20% of the blocks, that is, on average one block is left on the board at the end of each run. In contrast, with the RgTr policy all the blocks are grasped, resulting in a failure rate of 0%. Similarly, both the average number of attempts and the average number of collisions per block with the RgTr policy is also lower than the Rg policy, that is, 1.85 and 0.55 vs. 3.30 and 1.45. This difference in performance is justified by the fact that in the case of RgTr, once a collision occurs the regrasp policy ensures that the grasp attempt is successful. If the grasp position is sampled randomly, there will be a success chance of 1/4 for each grasping attempt. In contrast, with the touch feedback enabled, this chance jumps to 2.5/4 on average. As a consequence, the RgTr policy finds a successful grasp more quickly and thus grasps more blocks within the maximum five attempts limit. This experiment shows that sensing contacts outside the grasp closure offers an important feature to improve the success chance of a given grasp attempt.

**Table 4:** The table summarizes the percentage of failing to grasp blocks (failure rate), and the average number of attempts and collisions per block in all the grasping attempts (4 × 5).

**Policies**	**Failure rate**	**Avg. number of attempts per block**	**Avg. number of collisions per block**
Control	0%	1	0
Random grasp	20%	3.30	1.45
Random grasp + Touch informed regrasp	0%	1.85	0.55

*It can be noted that the Random grasp + Touch informed regrasp policy outperforms Random grasp in all three metrics, that is, obtains lower failure rate, less attempts, and less collisions*.

## 5. Discussion

**Figure 11:**
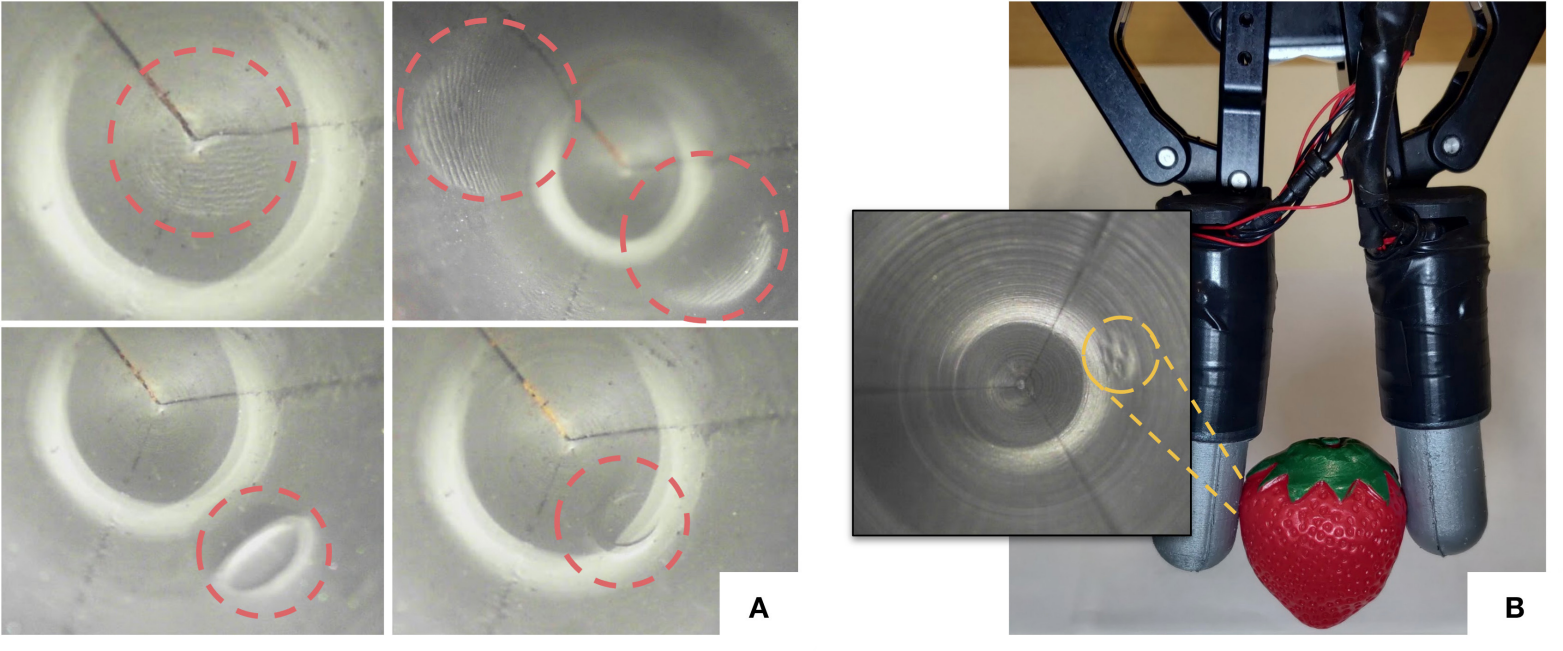
**(A)** Tactile images captured using our proposed GelTip sensor. From left to right, top to bottom: a fingerprint pressed against the tip of the sensor, two fingerprints on the sides, an open-cylinder shape being pressed against the side of the sensor, and the same object being pressed against the corner of the tip, all highlighted in red circles. **(B)** A plastic strawberry being grasped by a parallel gripper equipped with two GelTip sensors, with the corresponding imprint highlighted in the obtained tactile image (in gray scale).

The main aim of this work is to create an optical tactile sensor of all-around sensing for robotic grasping tasks, as illustrated in Figure 11. It can be seen that fine ridges of human fingerprints can be perceived in Figure 11A while the fine texture of a plastic strawberry can also be captured by the sensor in Figure 11B. Compared to the GelSight sensors (Li et al., 2014; Yuan et al., 2017), due to the sensor design of a finger shape, the light distribution throughout the sensor internal surface is no longer homogeneous. Specifically, a brightly illuminated ring can be observed near the discontinuity region (see Figure 2B). Shadows can also be observed in the bottom-left sample of Figure 11A when contacts of large pressure are applied, due to the placement of the camera and light sources. It may pose a challenge to geometry reconstruction using the Poisson reconstruction method (Li et al., 2014; Yuan et al., 2017; Romero et al., 2020) that builds a fixed mapping of pixel intensities to surface orientations and requires carefully placed RGB LEDs. In future research, convolutional neural networks could be used for geometry reconstruction of the GelTip sensor.

The new sensor geometry also introduces *ghost contacts* (detailed in section 4.1.3), resulted from projection of a contact onto the opposite side of the sensor. A deeper study on adjustment of the involved reflection coefficients and improved image analysis could mitigate this issue. The measurement of force fields using GelSight sensors using imprinted markers has been discussed in Yuan et al. (2017). We will also investigate adding such markers to the GelTip sensor in our future research.

## 6. Conclusions

In this paper, we propose a novel GelTip optical tactile sensor for all-around finger touch sensing. The GelTip sensor offers multiple advantages when compared against other camera-based tactile sensors, especially being able to capture high-resolution readings throughout its entire finger-shaped surface. The experiments show, for instance, that the sensor can effectively localize these contacts with a small error of approximately 5 mm, on average, and under 1 mm in the best cases. More importantly, the grasping experiments in the *Blocks World* environment show the potential of the all-around finger sensing in facilitating dynamic manipulation tasks. In our future research, we will introduce imprinted markers to the GelTip sensor to track the force fields. The use of the GelTip sensor in the manipulation tasks, such as grasping in cluttered environments, will also be of our interest.

## Data Availability Statement

The datasets generated for this study are available on request to the corresponding author.

## Author Contributions

DG contributed with the design of the sensor, paper writing and experiments. ZL contributed with initial exploratory experiments. SL contributed with writing and guidance. All authors contributed to the article and approved the submitted version.

## Conflict of Interest

The authors declare that the research was conducted in the absence of any commercial or financial relationships that could be construed as a potential conflict of interest. The handling editor is currently organizing a Research Topic with one of the authors SL.
