# Supplementary material for: Blocks World of Touch: Exploiting the Advantages of All-Around Finger Sensing in Robot Grasping
Source: Front Robot AI. 2020 Nov 19;7:541661. doi: 10.3389/frobt.2020.541661 (PMC7805632; doi:10.3389/frobt.2020.541661)
Supplement: Supplementary file 2 [file Data_Sheet_1.PDF]

Supplementary Material

1 Z COORDINATE DERIVATION

Bellow, the necessary steps to obtain the z component of the projective function m, that is introduced in the paper, Section 2. Due to the discontinuity in the sensor modeling, z can be obtained using S1, in the case of $(x' - c_x)^2 + (y' - c_y)^2 < (\frac{r\alpha}{d})^2$, else S2.

$$\begin{cases} x = (\frac{x'-c_x}{\alpha})z \\ y = (\frac{y'-c_y}{\alpha})z \\ x^2 + y^2 = r^2 \end{cases}$$

$$\Leftrightarrow \begin{cases} \\ [(\frac{x'-c_x}{\alpha})z]^2 + [(\frac{y'-c_y}{\alpha})z]^2 = r^2 \end{cases}$$

$$\Leftrightarrow \begin{cases} \\ (x' - c_x)^2 + (y' - c_y)^2 = (\frac{r\alpha}{z})^2 \end{cases}$$

$$\Leftrightarrow \begin{cases} \\ z = \sqrt{\frac{(r\alpha)^2}{(x'-c_x)^2+(y'-c_y)^2}} \end{cases} \tag{S1}$$

$$\begin{cases} x = (\frac{x'-c_x}{\alpha})z \\ y = (\frac{y'-c_y}{\alpha})z \\ x^2 + y^2 + (z-d)^2 = r^2 \end{cases}$$

$$\Leftrightarrow \begin{cases} [(\frac{x'-c_x}{\alpha})z]^2 + [(\frac{y'-c_y}{\alpha})z]^2 + (z-d)^2 = r^2 \end{cases}$$

$$\Leftrightarrow \begin{cases} \frac{(x'-c_x)^2}{\alpha^2}z^2 + \frac{(y'-c_y)^2}{\alpha^2}z^2 + \frac{\alpha^2(z^2-2zd+d^2)}{\alpha^2} = r^2 \end{cases}$$

$$\Leftrightarrow \begin{cases} (x'-c_x)^2z^2 + (y'-c_y)^2z^2 + (\alpha^2z^2 - \alpha^2 2zd + \alpha^2d^2) = r^2\alpha^2 \end{cases}$$

$$\Leftrightarrow \begin{cases} -\alpha^2 2d \cdot z + \alpha^2d^2 + [(x'-c_x)^2 + (y'-c_y)^2 + \alpha^2] \cdot z^2 = r^2\alpha^2 \end{cases}$$

$$\Leftrightarrow \begin{cases} a = (x'-c_x)^2 + (y'-c_y)^2 + \alpha^2 \\ b = -\alpha^2 2d \\ c = (d^2 - r^2)\alpha^2 \\ z = \frac{-b \pm \sqrt{-b^2-4ac}}{2a} \end{cases}$$

$$\Leftrightarrow \begin{cases} z = \frac{\alpha^2 2d + \sqrt{[-\alpha^2 2d]^2 - 4[(x'-c_x)^2 + (y'-c_y)^2][(d^2-r^2)\alpha^2]}}{2[(x'-c_x)^2 + (y'-c_y)^2 + \alpha^2]} \end{cases} \tag{S2}$$
